# A High-Fat-Diet-Induced Microbiota Imbalance Correlates with Oxidative Stress and the Inflammatory Response in the Gut of Freshwater Drum (*Aplodinotus grunniens*)

**DOI:** 10.3390/antiox13030363

**Published:** 2024-03-18

**Authors:** Miaomiao Xue, Pao Xu, Haibo Wen, Jianxiang Chen, Qingyong Wang, Jiyan He, Changchang He, Changxin Kong, Xiaowei Li, Hongxia Li, Changyou Song

**Affiliations:** 1 Wuxi Fisheries College, Nanjing Agricultural University, Wuxi 214081, China; 2 Key Laboratory of Freshwater Fisheries and Germplasm Resources Utilization, Ministry of Agriculture and Rural Affairs, Freshwater Fisheries Research Center, Chinese Academy of Fishery Sciences, Wuxi 214081, China

**Keywords:** high-fat diet, gut microbiota, physiological homeostasis, *Aplodinotus grunniens*

## Abstract

Lipids are critical nutrients for aquatic animals, and excessive or insufficient lipid intake can lead to physiological disorders, which further affect fish growth and health. In aquatic animals, the gut microbiota has an important regulatory role in lipid metabolism. However, the effects of a high-fat diet on physical health and microbiota diversity in the gut of freshwater drum (*Aplodinotus grunniens*) are unclear. Therefore, in the present study, a control group (Con, 6%) and a high-fat diet group (HFD, 12%) were established for a 16-week feeding experiment in freshwater drum to explore the physiological changes in the gut and the potential regulatory mechanisms of bacteria. The results indicated that a high-fat diet inhibited antioxidant and immune capacity while increasing inflammation, apoptosis and autophagy in gut cells. Transcriptome analysis revealed significant enrichment in immune-related, apoptosis-related and disease-related pathways. Through 16S rRNA analysis, a total of 31 genus-level differentially abundant bacterial taxa were identified. In addition, a high-fat diet reduced gut microbial diversity and disrupted the ecological balance of the gut microbiota (Ace, Chao, Shannon and Simpson indices). Integrated analysis of the gut microbiota combined with physiological indicators and the transcriptome revealed that gut microbial disorders were associated with intestinal antioxidants, immune and inflammatory responses, cell apoptosis and autophagy. Specifically, genus-level bacterial taxa in Proteobacteria (*Plesiomonas*, *Arenimonas*, *Erythrobacter* and *Aquabacteriumb*) could serve as potential targets controlling the response to high-fat-diet stimulation.

## 1. Introduction

Carbohydrates, lipids and proteins are the main nutrients for fish growth. However, the production of fish meal, the optimal protein resource for aquatic animals, is increasingly scarce worldwide, which strictly limits the development of aquaculture. Therefore, lipids and carbohydrates are widely applied as non-protein energy sources in diets to partially replace the protein content [1]. However, fish have a low tolerance to carbohydrates, and their ability to utilize energy from digestible carbohydrates is limited [2]. Therefore, lipids are considered to be the main non-protein energy source for fish [3], providing energy and essential fatty acids and acting as carriers of nutrients such as the fat-soluble vitamins A, D and K [4,5]. With the fast development of aquaculture, high-fat diets are widely utilized because they promote growth and protein retention [6]. However, excessive lipids can cause various metabolic diseases [7,8] such as metabolic disorders, fat accumulation, inflammation and endoplasmic reticulum stress [9,10,11], which can affect fish health.

The gut is not only a major site for digestion and nutrient absorption but also a key part of host defense [1,12] The fish gut is a complex ecosystem in which the gut microbiota is an important component of intestinal environment and plays a crucial role in enhancing host immunity, nutrient metabolism and digestion, as well as healthy growth and reproduction [13,14,15]. Many factors, such as species, growth stage and environment, can regulate the gut microbial community [16]. It is universally recognized that diet plays a key role in determining the composition of the gut microbiota and that dietary lipid levels influence the metabolic capacity of the gut microbial community [11,17]. However, as the freshwater drum is a newly domesticated aquatic animal, the effects of a high-fat diet on its gut and microbiota have not been studied. In this study, we hypothesized that a high-fat diet may induce immune and inflammatory responses in the gut and that alterations in the gut microbiota are in response to an imbalance in the physiological homeostasis of the gut.

It is well known that alterations in the lipid content of aquaculture feeds may affect multiple rather than individual genes and signaling pathways in fish [18,19,20], and transcriptomics provides an effective and rapid technique to study the molecular mechanisms of tissues and organs under different conditions [21]. In addition, 16S rRNA gene sequencing has become a major technique for studying bacterial communities [22]. Therefore, transcriptome and gut microbiota analyses can be used to understand changes in gut molecular regulatory networks and microbiota in animals on high-fat diets. The combined analysis of the two can further identify the role of key gut bacteria in the regulation of functional genes.

The freshwater drum (*Aplodinotus grunniens*) is native to North and Central America and is the only freshwater species in the genus *Aplodinotus* [23]. Freshwater drum have no intermuscular bones and possess a higher proportion of edible mass than most fish, with pleasant-tasting and nutritious flesh rich in proteins, amino acids and fatty acids, especially the unsaturated fatty acids DHA and EPA. The inclusion of freshwater drum can also improve the fish quality and processing of aquatic products [24]. Therefore, we introduced freshwater drum and conducted a great deal of related research. We conducted feed domestication and discovered potential regulatory mechanisms in freshwater drum [25], explored the mechanisms of intestinal adaptation to hypothermia [26] and identified the role of *miR-1/AMPK* in the regulation of glycolipid metabolism [24] as well as the role of the PPAR signaling pathway in the hypothermic regulation of lipid and amino acid metabolic homeostasis [27]. The above studies found that freshwater drum are suitable for nationwide culture and promotion. However, in recent aquaculture, we have discovered that a high-fat diet can adversely affect freshwater drums. Based on this finding, we evaluated the effects of high-fat diet on growth performance (condition factor (CF), viscerosomatic index (VSI) and hepatosomatic index (HSI)) and physiological homeostasis, and we explored the potential regulatory mechanisms in the liver of freshwater drum under lipid deposition [28]. However, limited information is known about the mechanisms that regulate the gut and the microbiota of freshwater drum. Therefore, in the present study, we assessed the effects of a high-fat diet on the gut antioxidant capacity, immunocompetence and inflammatory response as well as apoptosis and autophagy in freshwater drum. The interactions between gut bacteria and differentially expressed genes were also investigated. These results can reveal the effects of a high-fat diet on gut health and physiological homeostasis as well as the response mechanisms of the gut microbiota, which is important for the sustainability of freshwater drum as a resource.

## 2. Materials and Methods

### 2.1. Ethics Statement

The care and use of the animals followed the guidelines of the Animal Care and Use Committee of the Nanjing Agricultural University, China, and was approved under those guidelines (WXFC 2021-0006). All animal procedures were performed in accordance with the Guideline for the Care and Use of Laboratory Animals in China. The ethical situation in this experiment was identical to that in our previously published paper [26].

### 2.2. Experimental Animals and Experimental Design

Experiments were performed at the Freshwater Fisheries Research Center, Chinese Academy of Fishery Sciences. About 40,000 healthy freshwater drum with an average of 20.88 ± 2.75 g were transported randomly to two outdoor fish ponds (pond size 667 m^2^, about 20,000 fish per pond) for the experiment. During the experiment, the control group (Con, 6% fat) and high-fat diet group (HFD, 12% fat) were fed compounded diets four times a day for four months during the experimental period (Table S1). The feeding amount was 3~5% of body weight every day. The water was taken from an underground source and then fully aerated. The water quality was maintained as follows: DO > 6 mg/L, pH 7.2~7.8, NO_2_^−^ < 0.02 mg/L and NH_3_ < 0.05 mg/L.

### 2.3. Sample Collection

After 16 weeks of rearing experiments, fish were starved for 24 h to evacuate the residual feed in the intestine. Freshwater drum from the control (Con) and high-fat diet (HFD) groups were randomly selected (27 per group) from each pond and anesthetized with MS-222 (100 mg/L, tricaine methanesulfonate, Sigma, St. Louis, MO, USA) to collect samples. The sampled fish were dissected on ice to collect the gut tissue. The whole gut and contents were collected, immediately frozen in liquid nitrogen and stored at −80 °C for sequencing and RT-PCR analysis.

### 2.4. Biochemical Index Analysis

According to the manufacturer’s instructions, enzyme activity levels were measured with 10% gut homogenate supernatant. Specifically, a total of nine gut samples were selected and measured in duplicate for each group. A 0.1~0.2 g portion of each gut sample was isolated and homogenized in ninefold saline (*w*/*v*). Centrifugation (2500 rpm, 4 °C) was performed for 10 min to collect the supernatant for further measurement.

The analyzed antioxidant parameters included glutathione peroxidase (GPx), glutathione (GSH), catalase (CAT) and malondialdehyde (MDA). In detail, GPx was measured by the colorimetric method (Category No. A005-1-2), GSH by the microplate method (Category No. A006-2-1), CAT by the ammonium molybdate method (Category No. A007–1-1) and MDA by the thiobarbituric acid (TBA) method (Category No. A003-1-1). All kits were purchased from Nanjing Jiancheng Bioengineering Institute, Nanjing, China.

### 2.5. Enzyme-Linked Immunosorbent Assay Analysis

According to the manufacturer’s instructions, nine gut samples were selected from each group and rinsed with pre-cooled PBS (0.01 M, pH = 7.4) to remove residual contents. The gut samples were separated 0.1~0.2 g, combined with ice-cold PBS to form a 10% (*w*/*v*) tissue homogenate and centrifuged at 5000× *g* and 4 °C for 10 min to collect the supernatant. Specifically, we analyzed tumor necrosis factor alpha (TNF-α, Category No. ml07310), interleukin 6 (IL-6, Category No. ml0257510), immunoglobulin M (IgM, Category No. ml025819) and immunoglobulin G (IgG, Category No. ml823766) content by the double antibody sandwich ELISA method using commercial kits (Shanghai Enzyme-linked Biotechnology Co., Ltd., Shanghai, China).

### 2.6. RNA Extraction and De Novo High-Throughput Sequencing

According to the method described by Chen [24], three gut samples were selected from each group for transcriptome sequencing. Specifically, total RNA was isolated from gut tissues with a Trizol kit (Invitrogen, Carlsbad, CA, USA). Agilent 2100 and Nanodrop apparatuses (ThermoFisher Ltd., Waltham, MA, USA) were used to examine RNA quality, and high-quality RNA (1.8 < OD260/280 < 2.0, RNA integrity number (RIN) ≥ 1.8, 28S/18S ≥ 1.0) was processed with oligo (dT) to enrich mRNA. Next, the mRNA was randomly split into small fragments of about 300 bp using random primers. Then, cDNA was synthesized using commercial kits (NEB7530, New England Biolabs, Ipswich, MA, USA). Finally, de novo high-throughput sequencing was performed with an Illumina NovaSeq6000 (Majorbio Bio-pharm Technology Co., Ltd., Shanghai, China). The raw data were filtered with fastp (version 0.18.0) to obtain high-quality reads. Sequences were read using Trinity assembly and splicing as well as functionally annotated against the NR, Swiss Port, Pfam, KOG and GO databases. Transcripts with |log_2_fold change| > 1 and *p* < 0.05 were considered differentially expressed genes (DEGs). The enrichment analysis was performed with the Gene Ontology (GO) and Kyoto Encyclopedia of Genes and Genomes (KEGG) databases.

### 2.7. Microbial DNA Extraction and 16S Sequencing

In order to explore the diversity and composition of gut bacteria under the condition of a high-fat diet, six samples of gut contents were randomly selected for microbiome analysis according to the method described by Song [25]. First, an E.Z.N.A.^®^ Soil DNA kit (Omega Bio-Tek, Norcross, GA, USA) was used to extract microbial DNA [29]. The integrity of the obtained DNA was measured using 1% agarose gel electrophoresis and a Nanodrop ND2000 spectrophotometer (Thermo Scientific, Waltham, MA, USA). Isolated DNA concentrations were measured using a Quant-iT PicoGreen dsDNA Assay Kit (Invitrogen, Eugene, OR, USA) and a fluorometer and diluted to 20 ng/μL. Full-length 16S rRNA was amplified using primers 338 F (5′-ACTCCTACGGGAGGCAGCAG-3′) and 806 R (5′-GGACTACHVGGGTWTCTAAT-3′) by PCR. The PCR products from the same samples were mixed and detected using 2% gel electrophoresis, and the purified PCR products were recycled using an AxyPrepDNA Gel Extraction Kit (AXYGEN, Hangzhou, China). PCR products were quantified using a QuantiFluor™-ST Blue Fluorescence Quantification System (Promega, Madison, WI, USA). The purified PCR products were sequenced using the MiSeq platform (Illumina, San Diego, CA, USA).

PE reads obtained from Illumina sequencing were first spliced based on overlap relationships, while quality control and filtering of sequence quality were performed to arrive at the final valid data (fastq files). The RDP classifier Bayesian algorithm was used to analyze the taxonomy of the OTUs’ representative sequences at a 97% similarity level and to count the community species composition of each sample at the domain, kingdom, phylum, class, order, family, genus and species levels, respectively. Alpha diversity indices (Ace, Chao, Shannon and Simpson indices) of the samples were analyzed using Mothur 1.30.2 software. Beta diversity (weighted UniFrac and unweighted UniFrac distances) analysis was performed using QIIME software (version 1.9.1). The species abundance of the samples was counted at both the phylum and genus taxonomic levels, and the composition of the bacteria was analyzed.

### 2.8. Integrated Analysis between Key Genes and Bacteria

Differentially abundant bacteria were analyzed using Pearson correlation with gut-health-related genes (relevant to antioxidants, immunity, inflammation, autophagy and apoptosis) and DEGs in the transcriptome. In addition, DEGs and bacteria with *p* < 0.05 and *p* < 0.01 correlation were integrated for network interaction analysis in Cytoscape (version 3.9.0).

### 2.9. Transcriptional Expression Analysis by Real-Time PCR

Total RNA from nine gut samples in each group was extracted with RNAiso Plus reagent (Takara Co., Ltd., Dalian, China) and incubated with RNase-free DNase (Takara Co., Ltd., Dalian, China) to eliminate contaminating genomic DNA. The cDNA was synthesized from 1 µg of high-quality RNA (1.8 < OD260/280 < 2.0) with a PrimeScript™ RT reagent kit (TaKaRa, Dalian, China). Finally, RT-PCR was performed using a TB Green Premix Ex Taq^TM^ II (Tli RNase Plus) kit (TaKaRa, Dalian, China). The relative expression levels of genes were normalized to β-actin and further calculated using the 2^−ΔΔCT^ method. All the genes involved in this experiment were designed with Premier 5.0 based on the mRNA sequences obtained from the *A. grunniens* genome database in our laboratory and synthesized by Shanghai Generay Biotechnology Co., Ltd., Shanghai, China (Table S2).

### 2.10. Statistical Analysis

In this study, all data were analyzed using SPSS software (version 26.0) and presented as the mean ± standard error of the mean (SEM). Antioxidant parameters and ELISA data were analyzed in SPSS 26.0 with the independent-samples *t*-test. The 2^−∆∆CT^ method was used to calculate genes’ relative expression levels, followed by an independent-samples *t*-test. In data analysis, an independent-samples *t*-test was used when data were normally distributed and homoscedastic; otherwise, two independent-samples nonparametric tests (Mann-Whitney U) were used. Similarly, Pearson correlation analysis of genes and bacteria was performed using SPSS. Alpha diversity indices (Ace, Chao, Shannon and Simpson indices) were analyzed by one-way analysis of variance (ANOVA) followed by Tukey’s post hoc test using SPSS 26.0. *p* < 0.05 indicated a significant difference or correlation.

## 3. Results

### 3.1. A High-Fat Diet Inhibits Antioxidant Capacity in the Gut of A. grunniens

**Figure 1:**
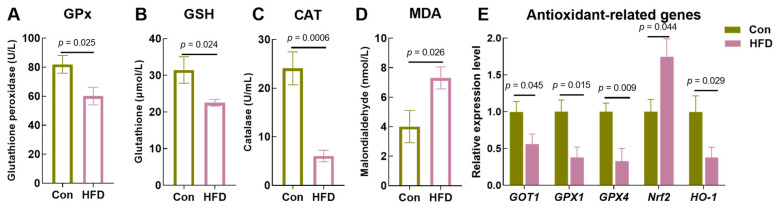
HFD inhibits antioxidant capacity in the gut of *A. grunniens*. (**A**), Glutathione peroxidase, GPx; (**B**), Glutathione, GSH; (**C**), Catalase, CAT; (**D**), Malondialdehyde, MDA; (**E**), Transcriptional expression of antioxidant-related genes. Results are indicated as the mean ± SEM, *n* = 9.

To explore the effects of a high-fat diet on the physiological homeostasis of the freshwater drum’s gut, we first assessed the antioxidant capacity. Results showed that the expression levels of GPx (*p* = 0.025), GSH (*p* = 0.024) and CAT (*p* = 0.0006) were significantly decreased in the HFD group compared to the Con group (Figure 1A,C). In contrast, the content of MDA was remarkably increased (*p* = 0.026, Figure 1D). In addition, RT-PCR results further revealed that the HFD reduced the expression of the antioxidant-related genes Glutamic-Oxaloacetic Transaminase 1 (*GOT1*, *p* = 0.045), glutathione peroxidase-1 (*GPX1*, *p* = 0.015), glutathione peroxidase-4 (*GPX4*, *p* = 0.009) and heme oxygenase 1 (*HO-1*, *p* = 0.029) compared to the Con. However, the HFD activated the transcription level of nuclear erythroid-related factor 2 (*Nrf2*, *p* = 0.044) (Figure 1E). These results indicate that an HFD inhibits gut antioxidant capacity, leading to physiological disorders in freshwater drum.

### 3.2. A High-Fat Diet Suppresses Immunocompetence and Induces Cellular Inflammation, Apoptosis and Autophagy

**Figure 2:**
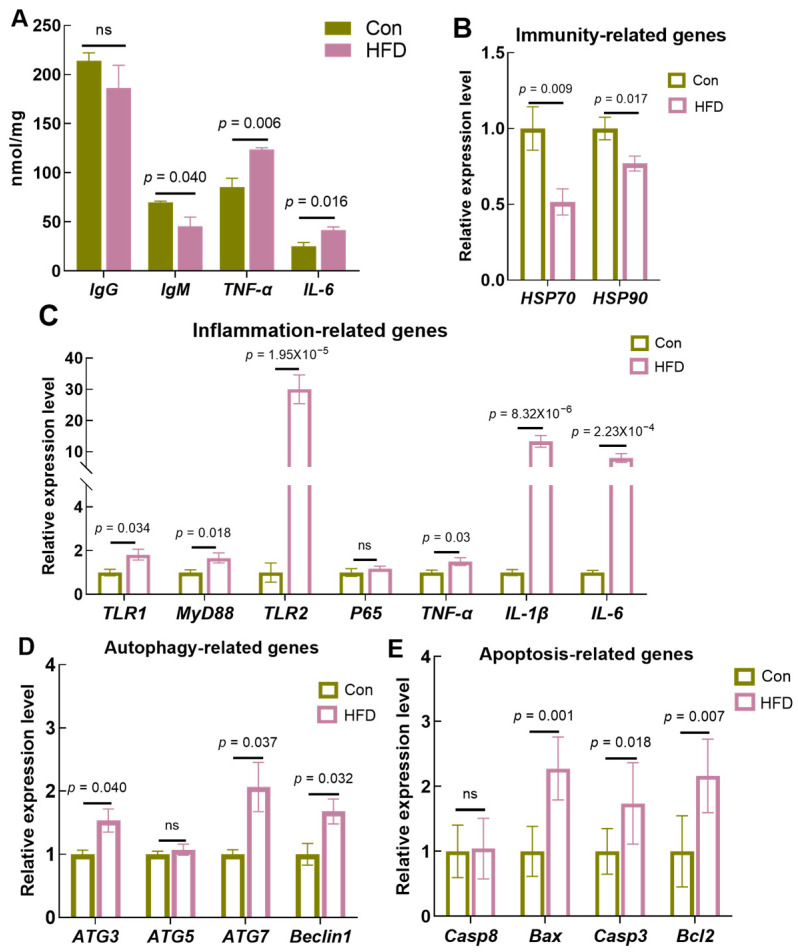
HFD suppresses immunity and induces cellular inflammation, apoptosis and autophagy in the gut of *A. grunniens*. (**A**), Enzyme-linked immunosorbent assay (ELISA) indicators; (**B**), Transcriptional expression of immunity-related genes; (**C**), Transcriptional expression of inflammation-related genes; (**D**), Transcriptional expression of autophagy-related genes; (**E**), Transcriptional expression of apoptosis-related genes. Results were indicated as mean ± SEM, *n* = 9. “ns” indicated no significant difference.

To determine whether physiologic disturbances resulting from high-fat diets caused immune and inflammatory responses, we evaluated the levels of immune and inflammatory factors. Enzyme-linked immunosorbent assays (ELISAs) showed decreasing trends in IgG (*p* > 0.05) and IgM (*p* = 0.04) in the gut; however, there were no significant differences in IgG after HFD feeding. Unexpectedly, *TNF-α* (*p* = 0.006) and *IL-6* (*p* = 0.016) levels were markedly increased after HFD feeding (Figure 2A). Meanwhile, RT-PCR results demonstrated that the immunity-related genes heat shock protein 70 (*HSP70*, *p* = 0.009) and heat shock protein 90 (*HSP90*, *p* = 0.017) were dramatically downregulated in freshwater drum receiving an HFD compared to the control group (Figure 2B). In addition, a high-fat diet increased the transcript levels of the inflammation-related genes toll-like receptor 1 (*TLR1*, *p* = 0.034), toll-like receptor 2 (*TLR2*, *p* = 1.95 × 10^−5^), myeloid differentiation protein-88 (*MyD88*, *p* = 0.018), interleukin-1 (*IL-1β*, *p* = 8.23 × 10^−6^) and interleukin-6 (*IL-6*, *p* = 2.23 × 10^−4^) compared to the Con. However, there was no significant difference in nuclear factor kappa B p65 (*P65*, *p* > 0.05) (Figure 2C).

Immune and inflammatory responses usually lead to cellular autophagy and apoptosis. Therefore, we examined the transcriptional expression of apoptosis- and autophagy-related genes. The results showed that HFD feeding significantly increased the mRNA expression of autophagy-related genes 3 and 7 (*ATG3*, *p* = 0.040; *ATG7*, *p* = 0.037) and *Beclin1* (*p* = 0.032) (Figure 2D). In addition, the expression levels of the apoptosis-related genes bcl2-associated X (*Bax*, *p* = 0.001), caspase 3 (*Casp3*, *p* = 0.018) and B-cell lymphoma-2 (*Bcl2*, *p* = 0.007) were significantly increased (Figure 2E). The above results indicate that a high-fat diet disrupts the physiological homeostasis of the gut, which further induces gut immune and inflammatory responses and increases cellular autophagy and apoptosis.

### 3.3. Gut Transcriptome Analysis of A. grunniens on a High-Fat Diet

**Figure 3:**
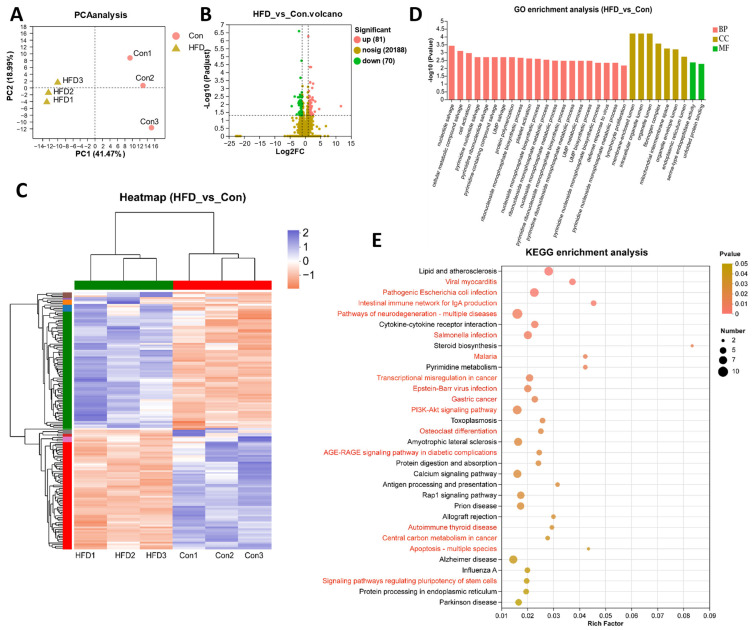
The transcriptome reveals that HFD dysregulates immunity and inflammation in the gut of *A. grunniens*. (**A**), PCA analysis between samples; (**B**), UniGene volcano plot; (**C**), Heatmap clustering of DEGs; (**D**), GO annotation of DEGs; (**E**), KEGG enrichment of DEGs (HFD vs. Con).

To reveal the potential regulatory mechanisms through which HFD feeding acts on the gut, we performed transcriptome analysis using high-throughput sequencing. Principal component analysis (PCA) showed that the samples in the Con and HFD groups were clustered into separate clusters, revealing clear differences between the transcriptome profiles of the Con and HFD groups (Figure 3A). A total of 151 differentially expressed genes (DEGs) were identified (|log_2_fold change| > 1, *p*-value < 0.05) compared with the Con group, of which 81 were significantly upregulated and 70 were downregulated (Figure 3B). Based on the expression levels, these DEGs were clustered into different subgroups on a heatmap (Figure 3C).

These DEGs were analyzed for GO enrichment, with a total of 135 GO items enriched, among which the top 30 items included 7 cellular components (CC), 21 biological processes (BP) and 2 molecular functions (MF) (Figure 3D, Table S3). Specifically, membrane-enclosed lumen (GO:0031974), intracellular organelle lumen (GO:0070013) and organelle lumen (GO:0043233) were the most significantly enriched GO items, suggesting that an HFD might affect gut organelles’ normal structure. KEGG enrichment was also utilized for DEG analysis (Figure 3E, Table S4). The results showed that DEGs were mainly enriched in the immune system (intestinal immune network for IgA production, antigen processing and presentation), disease and cancer (such as pathogenic Escherichia coli infection, salmonella infection, malaria, transcriptional misregulation in cancer, AGE-RAGE signaling pathway in diabetic complications, prion disease, autoimmune thyroid disease and central carbon metabolism in cancer), apoptosis in multiple species, signal transduction (PI3K-Akt signaling pathway, signaling pathways regulating pluripotency of stem cells) and metabolism (steroid biosynthesis and pyrimidine metabolism). The transcriptome results suggest that an HFD disrupts the immune system, generates inflammation and apoptosis and affects signaling transduction and metabolic systems in the gut.

### 3.4. Gut Microbiota Alternation of A. grunniens Fed with a High-Fat Diet

**Figure 4:**
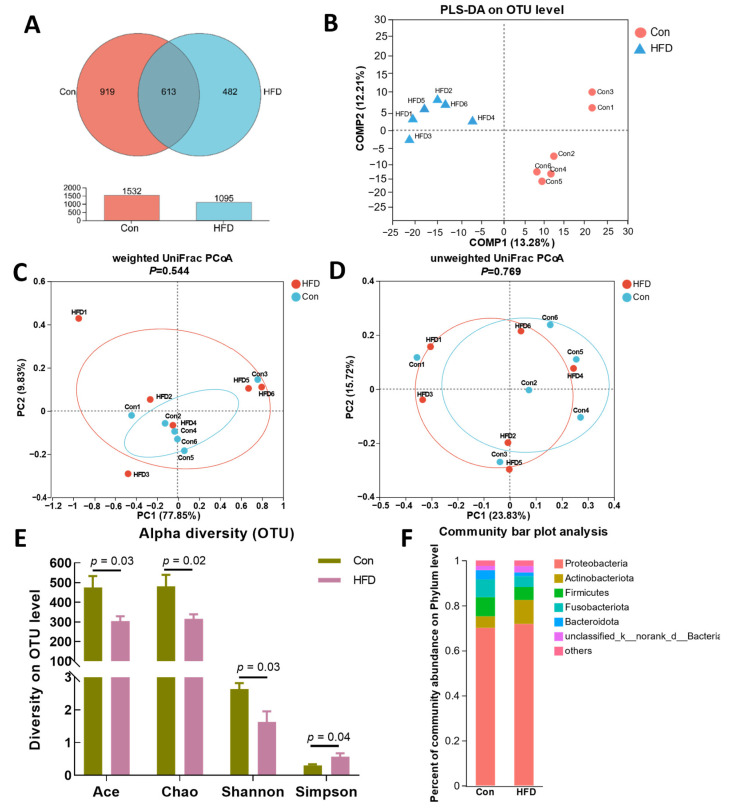
HFD-induced gut microbiome diversity alterations in *A. grunniens*. (**A**), Venn diagram analysis; (**B**), Partial least-squares discriminant analysis (PLS-DA) on the OTU level; (**C**), Weighted UniFrac index; (**D**), Unweighted UniFrac index; (**E**), Alpha diversity of the operational taxonomic units (OTUs); (**F**), Community bar plot analysis on the phylum level.

Gut health is tightly correlated with microbiota composition; therefore, we determined the composition of the microbial community by 16S rRNA gene amplicon sequencing. A total of 2014 operational taxonomic units (OTUs) were identified, corresponding to 41 phyla, 109 classes, 246 orders, 415 families, 798 genera and 1332 species (Table S5). Among them, a total of 1532 OTUs were identified in the Con group and 1095 in the HFD group, with co-enrichment of 613 OTUs (Figure 4A). Meanwhile, PLS-DA analysis of the samples revealed that the two groups clustered into different subgroups, indicating that HFD feeding affected the composition of OTUs in the gut (Figure 4B). The results of the beta diversity indices (weighted UniFrac and unweighted UniFrac) analyses showed that the differences in the diversity of the gut bacterial community between the HFD and Con groups were not significant (Figure 4C,D, *p* > 0.05). Ace, Chao, Shannon and Simpson’s analysis methods were applied to evaluate the alpha diversity of OTUs, and the results indicated that the OTUs exhibited a significant difference between the Con and HFD groups (Figure 4E, *p* < 0.05). In addition, gut microbiota composition was assessed by hierarchical clustering, and bacteria of the taxa Proteobacteria, Actinobacteriota, Firmicutes, Fusobacteriota, Bacteroidota and unclassified_k__norank__d__Bacteria were found to exhibit the richest diversity at the phylum level (Figure 4F).

### 3.5. Dominant Microbe Distribution in the Gut of A. grunniens Fed with a High-Fat Diet

**Figure 5:**
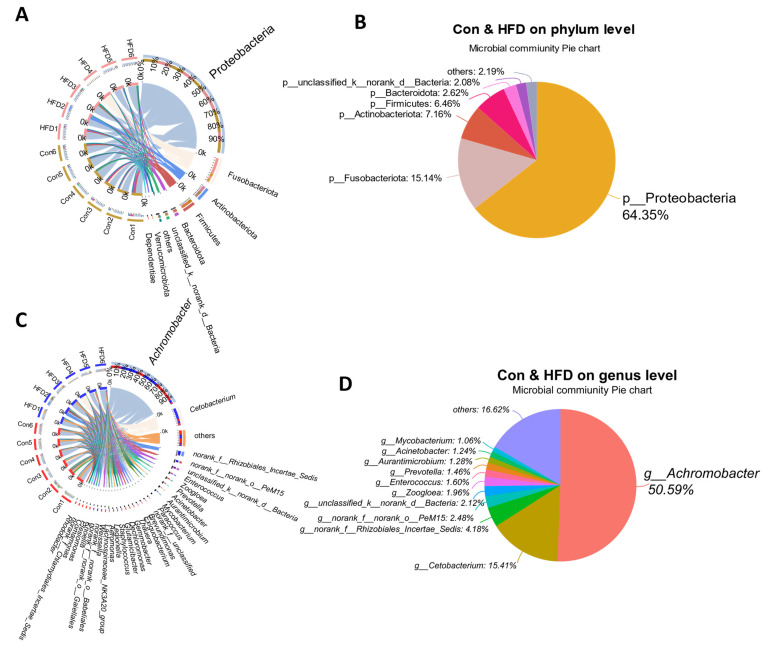
Dominant microbe distribution in the gut of *A. grunniens* fed with an HFD. (**A**), Circos samples and species relationships at the phylum level; (**B**), Species distribution pie chart at the phylum level; (**C**), Circos samples and species relationships at the genus level; (**D**), Species distribution pie chart at the genus level.

To further explore microbiota variation, we compared the distribution and proportions of major bacteria at the phylum and genus levels in all gut samples from freshwater drum. At the phylum level, a total of eight dominant phyla were identified, 6 of which had an average abundance >1%, including Proteobacteria (64.35%), Fusobacteriota (15.14%), Actinobacteriota (7.16%), Firmicutes (6.46%), Bacteroidota (2.62%) and unclassified_k__norank_d__Bacteria (2.08%) (Figure 5A,B). At the genus level, a total of 31 dominant genera were identified, 11 of which had an average abundance >1%, including *Achromobacter* (50.59%), *Cetobacterium* (15.41%), *norank_f__Rhizobiales_Incertae_Sedis* (4.18%), *norank_f __norank_o__PeM15* (2.48%), *unclassified_k__norank_d__Bacteria* (2.12%), *Zoogloea* (1.96%), *Enterococcus* (1.60%), *Prevotella* (1.46%), *Aurantimicrobium* (1.28%), *Acinetobacter* (1.24%) and *Mycobacterium* (1.06%) (Figure 5C,D). The above data indicate that Proteobacteria and *Achromobacter* are the most abundant colonizing bacteria at the phylum and genus levels in the gut of freshwater drums under HFD feeding.

### 3.6. Microbial Comparison and Phylogenetic Tree Analysis

**Figure 6:**
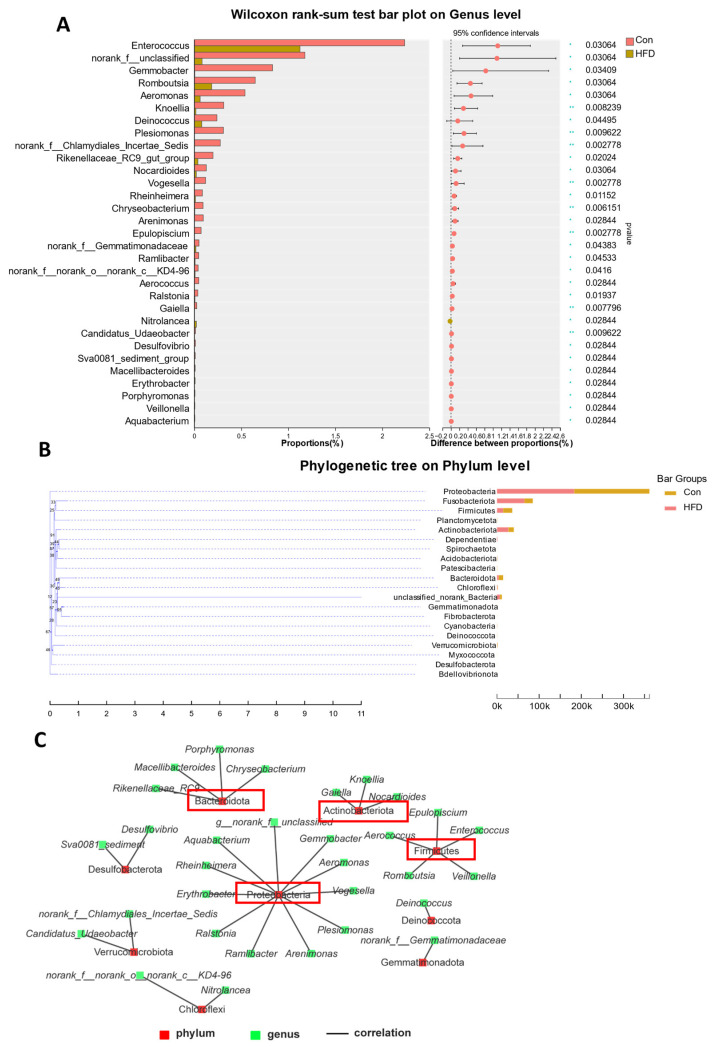
Microbial comparison and phylogenetic analysis in the gut of *A. grunniens* fed with an HFD. (**A**), Differentially abundant colonizing microbial communities on the genus level; (**B**), Classification of genus-level differentially abundant microorganisms and corresponding phyla; (**C**), Phylogenetic analysis on the phylum level. Red boxes represent phylum with more classification at the genus level.

Based on the above findings, we further analyzed the bacteria with differential abundance between the two groups. At the genus level, a total of 31 differentially abundant bacteria were identified, and all of them exhibited a significant difference in the HFD group (*p* < 0.05, Figure 6A). With the classification of these bacteria, we found that they belonged to nine phyla, including Firmicutes (five different bacteria), Proteobacteria (eleven), Actinobacteriota (three), Deinococcota (one), Verrucomicrobiota (two), Bacteroidota (four), Gemmatimonadota (one), Chloroflexi (two) and Desulfobacterota (two). Interestingly, the most enriched phylum was Proteobacteria (Figure 6C). In addition, phylogenetic analyses of these dominant bacteria were conducted, which indicated that Proteobacteria are evolutionarily distant from other bacteria at the phylum level (Figure 6B).

### 3.7. Correlation Analysis of Gut Antioxidant, Immune, Inflammatory, Autophagy and Apoptosis Genes with Gut Bacteria

**Figure 7:**
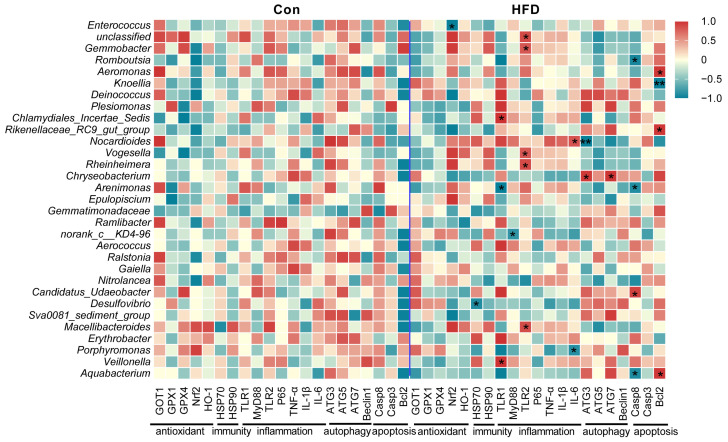
Correlation analysis between gut microbe abundance and gut health factors. Data were analyzed using Pearson correlation analysis with SPSS 26.0. * represents a statistical difference (*, *p* < 0.05; **, *p* < 0.01).

To investigate whether gut bacteria are involved in the regulation of gut health and physiological homeostasis, we subjected 31 differential abundant genus-level bacteria to Pearson correlation analysis with antioxidant, immune, inflammatory, autophagy and apoptosis genes as shown in Figure 7. We found that some gut bacteria are involved in the regulation of gut health after consumption of a high-fat diet. *Enterococcus* was significantly associated with antioxidant gene (*Nrf2*, *p* < 0.05). *Desulfovibrio* was significantly correlated with an immune gene (*HSP70*, *p* < 0.05). *Chlamydiales_Incertae_Sedis*, *Arenimonas*, *Veillonella*, *Norank*, *Unclassified*, *Gemmobacter*, *Gemmobacter*, *Rheinheimera*, *Macellibacteroides*, *Nocardioides* and *Porphyromonas* were significantly related to inflammatory genes (*TLR1*, *MyD88*, *TLR2*, *IL-6*, *p* < 0.05), respectively. *Nocardioides* and *Chryseobacterium* were significantly correlated with autophagy genes (*ATG3*, *ATG7*, *p* < 0.05). *Romboutsia*, *Arenimonas*, *Candidatus_Udaeobacter*, *Aquabacterium*, *Aeromonas*, *Knoellia* and *Rikenellaceae_RC9_gut_group* were significantly associated with apoptotic genes (*Casp8*, *Bcl2*, *p* < 0.05), respectively. The above results demonstrate that gut microbial dysbiosis on a high-fat diet is closely related to gut health and physiological homeostasis.

### 3.8. Integrated Analysis between DEGs and Bacteria of A. grunniens on a High-Fat Diet

**Figure 8:**
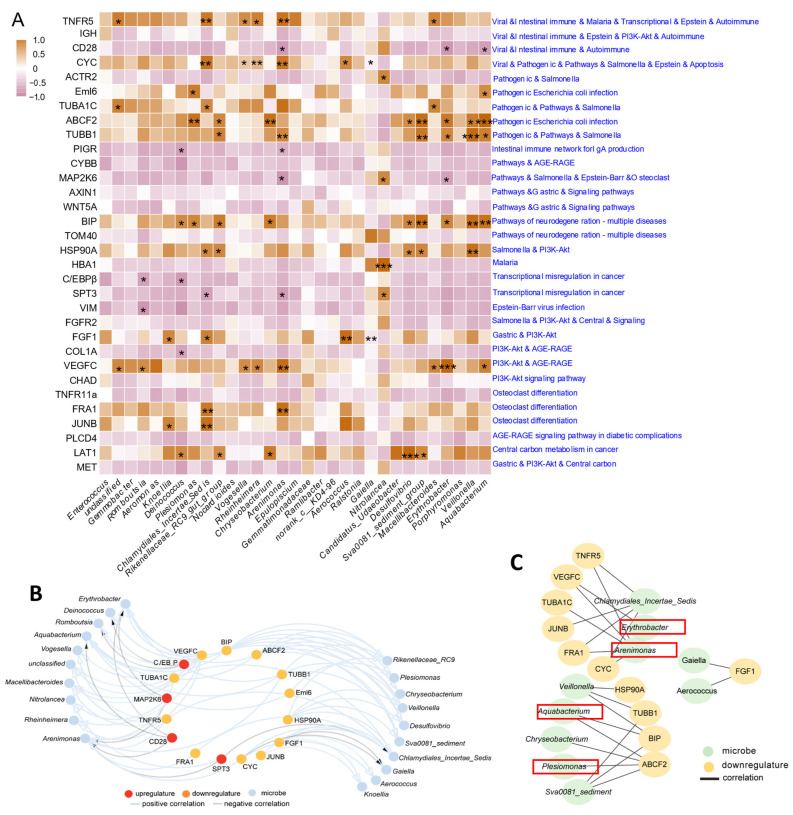
Integrated analysis of DEGs and bacteria on an HFD in *A. grunniens*. (**A**), Pearson correlation analysis between key bacteria at the genus level and enriched genes; (**B**), Interacting regulatory network between bacteria and genes with correlative differences (*p* < 0.05). In the network, red nodes represent upregulated DEGs, orange nodes represent downregulated DEGs, blue nodes represent bacteria, blue lines indicate positive correlations and grey lines indicate negative correlations. (**C**), Interacting regulatory network between bacteria and genes with correlative differences (*p* < 0.01). In the network, yellow nodes represent downregulated DEGs, green nodes represent bacteria and all lines indicate positive correlations. Red boxes represent bacteria at the level of the genus Proteobacteria. * Refers to a significant correlation between bacteria and genes (*, *p* < 0.05; **, *p* < 0.01; ***, *p* < 0.001).

The integrated network analysis between DEGs and bacteria revealed potential mechanisms for gut regulation through HFD feeding. Pearson correlation analysis combining DEGs in 16 key pathways (immune, inflammatory, apoptotic and metabolic disease-related pathways) and 31 genus-level differentially abundant bacteria revealed significant correlations between 20 bacteria and 16 DEGs (*p* < 0.05, Figure 8A,B). Furthermore, we found extremely significant correlations between ten bacteria and eleven DEGs (*p* < 0.01, Figure 8C), among which four belonged to Proteobacteria, including *Plesiomonas*, *Arenimonas*, *Erythrobacter* and *Aquabacterium*. The above findings demonstrate that bacteria in the phylum Proteobacteria may be key factors in regulating gut health.

### 3.9. Expression Validation of Key Genes

**Figure 9:**
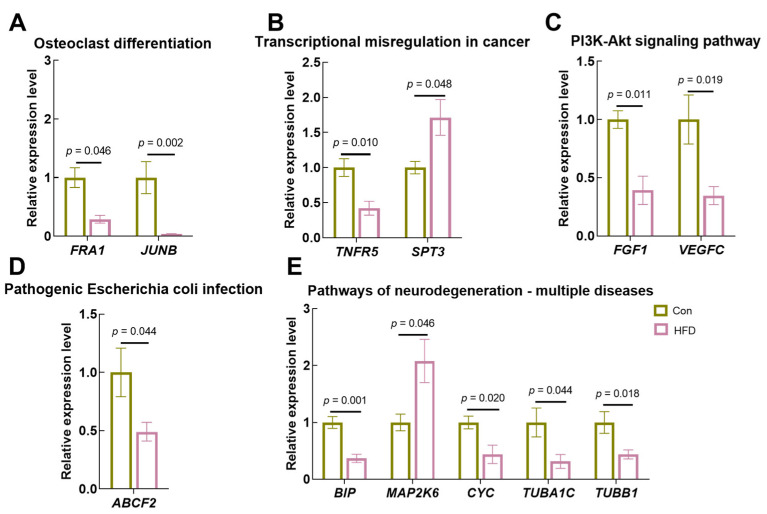
Transcriptional expression of key genes under HFD stimulation. (**A**), Transcriptional expression of genes related to protein digestion and absorption; (**B**), Transcriptional expression of genes related to transcriptional misregulation in cancer; (**C**), Transcriptional expression of genes related to the PI3K-Akt signaling pathway; (**D**), Transcriptional expression of genes related to pathogenic Escherichia coli infection; (**E**), Transcriptional expression of genes related to pathways of neurodegeneration—multiple diseases. Results are indicated as mean ± SEM, *n* = 9.

Next, we assessed the reliability of the transcriptome sequencing results using RT-PCR. Based on the integrated analysis between DEGs and bacteria, 12 DEGs were identified as having significant correlations with gut bacteria. These genes belong to four pathways, including protein digestion and absorption (Figure 9A, Fos-related antigen (*FRA1*, *p* = 0.020)), transcription factor jun-B (*JUNB*, *p* = 0.020)), transcriptional dysregulation in cancer cells (Figure 9B, tumor necrosis factor receptor superfamily member 5 (*TNFR5*, *p* = 0.010)), transcription initiation protein SPT3 homolog (*SPT3*, *p* = 0.048)), the PI3K-Akt signaling pathway (Figure 9C, Putative fibroblast growth factor 1 (*FGF1*, *p* = 0.011)), vascular endothelial growth factor C (*VEGFC*, *p* = 0.019), pathogenic Escherichia coli infection (Figure 9D, ATP-binding cassette sub-family F member 2 (*ABCF2*, *p* = 0.033)) and pathways of neurodegeneration—multiple diseases (endoplasmic reticulum chaperone BiP isoform (*BIP*, *p* = 0.001), dual specificity mitogen-activated protein kinase kinase 6 isoform X1 (*MAP2K6*, *p* = 0.020), cytochrome c (*CYC*, *p* = 0.020), tubulin alpha-1C chain isoform X2 (*TUBA1C*, *p* = 0.044), tubulin beta-1 chain-like (*TUBB1*, *p* = 0.018)) (Figure 9E). These data not only demonstrate the reliability of the results of differential gene expression analyses based on transcriptome sequencing data but also demonstrate that HFD affects gut health, cellular differentiation and signaling functions.

## 4. Discussion

Excessive lipid accumulation can have a serious impact on fish health and is one of the greatest challenges for aquaculture [17]. Our previous studies have shown that while an HFD promotes the growth of freshwater drum to some extent, excessive fat content produces more harm than good [28]. For example, HFD feeding led to liver lipid deposition in freshwater drum, along with decreased antioxidant capacity, and resulted in lipid metabolism disorders in the liver [28]. In fish, several studies have focused on microbial regulation and the contribution of the gut microbiota to host growth, immunomodulation and metabolism [30]. Therefore, based on the above studies, we further investigated the effects of an HFD on gut health and physiological homeostasis as well as on the ecological balance of microbial communities.

Like all aerobic organisms, fish are susceptible to reactive oxygen species (ROS). In addition to the organism’s inherent capacity for antioxidant defense, fish antioxidant defense is also dependent on feeding behavior and nutritional factors [31]. It has been shown that fish fed with a high-fat diet produce excessive amounts of ROS [32], while excessive levels of ROS can lead to lipid peroxidation, resulting in the production of large amounts of MDA [33]. MDA is a biomarker for assessing lipid peroxidation and can indirectly reflect the extent of free radical attack in fish [34,35]. In the current study, MDA content was markedly increased with HFD feeding, indicating that a high-fat diet leads to lipid peroxidation in the gut. In addition, organisms rely on antioxidant protection systems to prevent oxidative damage from HFD-induced stress [36]. GPx converts superoxide into harmless hydroxyl compounds and water, preventing their oxidation and the formation of dangerous free radicals [37]. GSH plays the role of a scavenger by oxidizing to GSSG [38]. CAT is the first line of the enzymatic antioxidant system [39]. The antioxidant system protects freshwater drum from oxidative stress. However, the production of oxidized substances that cannot be completely eliminated by the antioxidant system leads to the inhibition of antioxidant effects. The inhibition of the antioxidant enzyme system comprising CAT activity, GPx and GSH content further supports the conclusion that an HFD induces oxidative stress in the gut of freshwater drum. The same results of reduced antioxidant enzyme activity in aquatic animals fed a high-fat diet have also been reported in previous studies [40,41,42]. Importantly, an HFD also suppressed the transcript levels of antioxidant-related genes (*GOT1*, *GPX1* and *GPX4*). *GOT1* is essential for redox homeostasis and procreation in cells [43]. Activation of *GPX1* and *GPX4* mediates antioxidant processes [44,45]. Importantly, HFD-induced stress activates *Nrf2* signaling. As a key regulator of the antioxidant response, *Nrf2* protects cells from apoptosis when the organism is subjected to oxidative stress [46,47,48]. We found that an HFD activated *Nrf2* to enhance gut oxidative stress resistance. In particular, *HO-1* exerts potent antioxidant and anti-apoptotic effects [49]. Therefore, *Nrf2* may play a regulatory role with key genes of lipid peroxidation at the transcriptional level to alleviate the side effects of lipid peroxidation to some extent.

The ongoing development of oxidative stress and a decline in antioxidant capacity will inevitably affect immune capacity and exacerbate the inflammatory response. Immunoglobulins play an essential role in combating microbial invasion in fish [50]. The results showed that HFD feeding reduced immunoglobulin (IgG and IgM) levels. In addition, heat shock proteins (HSPs) are involved in cellular antioxidant and innate immune responses [26]. Transcript levels of *HSP70* and *HSP90* were dramatically decreased under HFD feeding, which may be attributable to oxidative stress caused by HFD as well as suppression of antioxidant capacity, which further compromises the gut immune system. To explore the possible effects of an HFD on gut inflammation, typical inflammatory parameters (*TNF-α* and *IL-6*) were used to assess gut inflammation. *TNF-α* is an inflammatory cytokine that has been extensively studied in fish [51]. *IL-6* plays a central role in host defense, which can alleviate inflammation and trigger an immune response under acute inflammation [52]. Both ELISA and RT-PCR results showed that *TNF-α* and *IL-6* increased significantly after HFD feeding. Specifically, the expression levels of other pro-inflammatory cytokines such as *TLR1*, *TLR2*, *MyD88*, *IL-1β* and *NF-κB* were all dramatically increased. These results indicate that excessive lipid intake induces a gut inflammatory response in freshwater drum. Autophagy has an important role in cellular self-protection and maintenance of cellular physiological homeostasis [53]. Additionally, autophagy is regulated by ROS signaling and oxidative stress [54]. In this study, the autophagy-related genes *ATG3*, *ATG5*, *ATG7* and *Beclin1* (a biomarker of autophagy) showed upregulation, indicating that HFD may disrupt the physiological homeostasis of the gut. This may further support the fact that excessive lipid intake causes oxidative stress through increased ROS levels which in turn leads to oxidative damage. To maintain normal physiological functions, the organism removes unnecessary or abnormal cells through apoptosis [26]. We discovered that an HFD activates apoptotic genes (*Casp8*, *Casp3*, *Bax*, *Bcl2*) in the gut, demonstrating that apoptosis is a key response mechanism for maintaining homeostasis in the body under a high-fat diet. Moreover, powerful evidence shows that inhibition of antioxidant capacity also promotes apoptotic responses [55,56].

Based on the above studies, we further revealed the effects of a high-fat diet on intestinal molecular regulation and microbial homeostasis using transcriptome and 16S rRNA gene sequencing. The transcriptome sequencing results showed that DEGs were mainly enriched in pathways associated with intestinal metabolic disorders, inflammation and even disease, suggesting that an HFD disrupts the immune system, generates inflammation and apoptosis and affects signaling and metabolic systems in the gut. This further confirms the negative impact of a high-fat diet on the gut. In addition, diet can influence interactions between the host and the microbial community, leading to changes in the structure of the gut microbiota [57]. The gut microbiota plays an important function in fish health by stimulating the immune system and aiding nutrient absorption [1]. It is well known that the external environment, such as the living temperature, various external stress, dietary intake and bacterial or viral infection, can disrupt the abundance and diversity of the gut bacteria in fish, thereby strongly affecting the health of the host [58]. In the present study, although the HFD group showed some degree of similarity in beta diversity and no non-significant differences compared to Con, the alpha diversity index was significantly lower, suggesting that HFD feeding has an impact on gut microbial abundance and diversity. Analysis of the dominant bacteria revealed that Proteobacteria and *Achromobacter* were the most abundant at the phylum and genus levels, respectively. Through Pearson correlation analysis, we found that some of the 31 different genus-level bacteria were involved in the regulation of gut antioxidant function, immunity, inflammation, autophagy and apoptosis. In addition, analysis of genus-level differentially abundant bacteria and interaction analyses with DEGs in 16 pathways indicated that *Plesiomonas*, *Arenimonas*, *Erythrobacter* and *Aquabacteriumb* under Proteobacteria were significantly associated with gut disease, cell differentiation and signaling DEGs. Based on the above findings, we speculate that gut bacteria, especially key genera in the phylum Proteobacteria, are collectively involved in the regulation of gut inflammation and metabolism under a high-fat diet in freshwater drum.

Studies have shown that the phylum Proteobacteria is one of the dominant phyla of the gut microbiota [59,60], associated with intestinal microbiota instability, and an increase in its abundance is one of the potential diagnostic criteria for ecological disorders and diseases [61,62]. Proteobacteria have also been used in previous studies to act as a “microbiological signature” of disease [63]. We found that although overall and genus-level microbial abundance tended to decrease under HFD feeding, the abundance of bacteria in the particular phylum Proteobacteria tended to increase. 

Studies have demonstrated that *Plesiomonas* is one of the core gut bacteria in zebrafish [64]. In addition, as a pathogenic bacterium, *Plesiomonas* has been shown to be associated with diseases in fish [65,66]. In the present study, HFD feeding was found to reduce the abundance of *Plesiomonas*. It was hypothesized that a high-fat diet would disturb the balance of the microbial community and may further induce gut diseases, which is similar to the findings in HFD-fed zebrafish [67]. Studies have shown that *Aquabacterium* has been detected in the insect gut and the water column [68,69,70], with especially large quantities being present in the water column [71]. Recent studies have identified *Aquabacterium* as the dominant microbe in the gill mucosa of *Salmo salar* [72]. Other studies have revealed that it may be a commensal bacterium that plays an important role in SVCV infections [59], but its exact function is not yet known. We hypothesize that the reduced abundance of *Aquabacterium* after HFD feeding may be associated with immune and inflammatory responses in the gut, but more studies are needed to provide evidence. Interestingly, previous studies have shown *Arenimonas* to be an environmentally relevant microbe, frequently isolated in iron ore and sewage, and have not described adverse effects on fish [73,74]. However, a reduced abundance of *Arenimonas* in the gut of zebrafish on a high-fat diet with added sea buckthorn was recently found [75]. Unusually, the opposite result was found in our study. Thus, the function of *Arenimonas* and the role it plays in relation to the fish gut deserve further investigation. Much of the research on *Erythrobacter* has focused on its utilization of light energy [76]. In addition to this, *Erythrobacter* was found to be present as a dominant microbial genus in the water column of *Penaeus vannamei* aquaculture [77], and the relative abundance of *Erythrobacter* was found to be increased in *Pandalus platyceros* on a high-fishmeal diet [78]. In the present study, the abundance of *Erythrobacter* was reduced. The inconsistency of the results of various studies indicates that diet-induced changes in the gut microbiota may depend on species and the health conditions of the fish itself. It has been shown that the functions of the gut microbiota are dynamic [79], which means that the same function can be performed by several bacteria. For the above bacteria whose specific functions are unknown, more research is needed to determine whether there is a symbiotic interaction between them to regulate the health of the gut. However, it is certain that important bacteria in the phylum Proteobacteria are important microbiota for the response of freshwater drum to metabolic disorders, inflammation and disease caused by a high-fat diet.

## 5. Conclusions

The present study indicates that oxidative stress induced by a high-fat diet in freshwater drum (20.88 ± 2.75 g) suppresses the antioxidant capacity of the gut, causing immune and inflammatory responses, as well as cellular autophagy and apoptosis. A high-fat diet leads to reduced abundance in the overall abundance of gut microbiota, some of which are strongly associated with intestinal immunity and the inflammatory response, cell apoptosis and autophagy. Importantly, genus-level bacterial taxa in Proteobacteria are involved in the regulation of gut health and physiological homeostasis.

## Data Availability

The data presented in this study are available in the article and Supplementary Materials.

## Supplementary Materials

The following supporting information can be downloaded at: https://www.mdpi.com/article/10.3390/antiox13030363/s1, Table S1: Formulation and proximate composition of experimental diets; Table S2: Primers and sequences referred to in the experiment; Table S3: Transcriptome GO enrichment analysis statistics for the high-fat diet experiment; Table S4: Transcriptome KEGG enrichment analysis statistics for the high-fat diet experiment; Table S5: Gut microbiota composition of freshwater drum on a high-fat diet.
