# Supplementary material for: A High-Fat-Diet-Induced Microbiota Imbalance Correlates with Oxidative Stress and the Inflammatory Response in the Gut of Freshwater Drum (Aplodinotus grunniens)
Source: Antioxidants (Basel). 2024 Mar 18;13(3):363. doi: 10.3390/antiox13030363 (PMC10967924; doi:10.3390/antiox13030363)
Supplement: Supplementary file 1 [file antioxidants-13-00363-s001.zip › Tables S1 and S2.pdf]

Table S1 Formulation and proximate composition of experimental diets

Ingredient/%	6% (Con)	12% (HFD)	Nutritional value (%, dry matter)	6% (Con)	12% (HFD)
Fish meal	55	55	Dry matter, DM	87.001	88.216
Vital wheat gluten	4	4	Crude protein, CP	45.131	45.131
Soybean protein concentrate	6	6	Ether extract, EE	6.07	12.048
α-Starch	18.5	5	Nitrogen-free extract, NFE	6.513	6.5435
Carboxymethylcellulose	0	7.4	Crude fat, CF	0.598	0.598
Fish oil	1	7.1	Ash	14.275	14.3055
Saccharomyces cerevisiae	3	3	Ca	3.2861	3.2861
Squid meal	2	2	P	2.16595	2.1646
codium humate	2	2	Total P	2.12715	2.1231
Sodium butyrate	1	1	Lysine	3.1891	3.1891
Ethoxyquin	0.5	0.5	Methionine+ccysteine	1.188	1.188
Ccholine chloride (50%)	1	1	Methionine	1.1874	1.1874
ccitamin premix[a]	1	1	Threonine	1.7298	1.7298
Mineral premix[b]	1	1	Arginine	2.5318	2.5318
Ccalcium dihydrogen Phosphate	2	2	Fe	203.27	203.27
Attapulgite	2	2	Gross ccnergy[c]	15.702	15.72444
Total	100	100			

Note: [a] Mineral content per kg dietc: $FeSO_4 \cdot 7H_2O$, 250 mg; $CuSO_4 \cdot 5H_2O$, 20 mg; $ZnSO_4 \cdot 7H_2O$, 220 mg; Na_2SeO_3, 0.4 mg; $MnSO_4 \cdot 4H_2O$, 70 mg; $CoCl_2 \cdot 6H_2O$, 1 mg; KI, 0.26 mg.

[b] Vitamin content per kg dietc: vitamin A, 9000 IU; vcitamin B1, 3.2 mg; vcitamin B2, 10.9 mg; vitamin B5, 20 mg; vcitamin B6, 5 mg; vcitamin B12, 0.016 mg; vitamin C, 50 mg; vcitamin D, 2000 IU; vcitamin E, 45 mg; vitamin K3, 2.2 mg; nciacin, 28 mg; fcolic acid, 1.65 mg; pantothenate, 10 mg; choline, 600 mg.

[c] Energy, calculated by using standard physiological fuel values of 37.7, 16.7, and 16.7 kJ/cg for proteins, lipidcc and carbohydrates, respectively.

Table S2 Primers and sequences referred cin the experiment

Accession No.	Gene	Primer sequence (5' — 3')	Amplification efficiency	Amplification size (bp)
XP_010740051.1	*GOT1*	F: AGAGCGTTGCACTACCAGTC<br>R: AAGTCACGCAGACAGAGTCG	94.26	139
XP_010743590.2	*GPX1*	F: TCCCATCCAACAACTTCCAC<br>R: TTCATCAGATACCCTCCTCA	104.26	125
ATX74747.1	*GPX4*	F: TGCTCAAGCGGGAGACT<br>R: AGCCCAGGATGCGTAAA	106.62	212
TKS67183.1	*Nrf2*	F: GGAGCGTCTGCTGAGTGA<br>R: AAGATGCTGCCGTTGGTT	108.07	168
KAE8300034.1	*HO-1*	F: GCTTACACCCGCTACCTC<br>R: GCAAAGAACGACAGACCC	94.26	104
XP_008328442.1	*β-actin*	F: AGGCTGTGCTGTCCCTGTAT<br>R: GCTGTGGTGGTGAAGGAGTAG	102.08	127
AKS36893.1	*HSP70*	F: ACCACCATTCCCACCAAG<br>R: CGACAGCGGAGACATTCA	105.87	223
AFK32353.1	*HSP90*	F: TGAACTTTGTCCGTGGTG<br>R: CTGTGAGGGAGGTCGTCT	94.17	291
AKJ66261.1	*TLR1*	F: AGATTATCGCTGTGCCTAC<br>R: TTTGCTCTGGTCCATTTC	98.38	204
XP_010751574.1	*MyD88*	F: GACTGGATGGTCGTTGC<br>R: GCTCGGACTTCTTCTTCA	92.79	244
AHB51066.1	*TLR2*	F: GGAGAAACCAGTGGGTCAAG<br>R: CAACAGAATGGCGACAAATAG	108.97	103
XM_019270684.2	*P65*	F: GTGGGAGGAGGAGTTTGA<br>R: CATAGATGGGCTGCGATA	112.37	113
XP_010735433.1	*TNF-α*	F: ATCGGCGTGCTGTTCAA<br>R: GCGACCGTGGGATTTAG	103.89	219
AQR55700.1	*IL-1β*	F: GCTGAACCTCAGTACCCTTGT<br>R: GAAGTTTCGGTGGCGTCT	92.53	193
XP_010733055.2	*IL-6*	F: AAGACTGCCCTTCCAACTAC<br>R: ATCAGATTGTCCCGCTCA	91.91	112
KY689036.1	*Casp8*	F: GGAGAACCGACTGGAGGAA<br>R: TGTAGATGGAGCCTGTGGAAG	83.56	134
XM_010749348.3	*Bax*	F: GAGGTGGTGGAACATCTGCT<br>R: TTGGTGGTCAGTGCCTTGTA	97.42	209
KF738809.1	*Casp3*	F: CTGCTACGCCTCGTTTGTCT<br>R: TCAGCTTCCACAGGGATCTT	107.57	240
XM_010739224.3	*Bcl2*	F: CCCAAGACGGGTTGTGAT<br>R: CCATATTGCCCTGCAAGTAG	110.28	224
XP_007542097.1	*ATG3*	F: GGTAGAAACTAAAGCCAAAG<br>R: CATATCCAAACAGCCAGA	107.33	156
XP_027142857.1	*ATG5*	F: GGAGGAGATGTGGTTCG	92.21	134

Accession No.	Gene	Primer sequence (5' — 3')	Amplification efficiency	Amplification size (bp)
		R GGAGGAGATGTGGTTCG		
TMS19022.1	*ATG7*	F: TGACCCACTTGGTTTGCC<br>R: TGGTGTTATACAGCGTTCC	107.28	137
XP_010744182.3	*Beclin-1*	F: ACATCACGGAGAACGAA<br>R: TCTGGCAGTACCGCATC	94.77	189
XP_027147112.1	*CYC*	F: AGGCATCATTTGGGACGAGG<br>R: GGCTCGTTCGGTCTTCTTCT	108.54	200
XP_019124562.1	*MAP2K6*	F: AGATGTGAAGCCCTCCAACG<br>R: AGCCTTTCTGGTTCGTCTCG	99.17	195
XP_036959003.1	*TUBA1C*	F: CCATCACTGCTTCCCTTCGT<br>R:CGGGGATATGGCACCAAGTT	107.86	163
XP_028313877.1	*TUBB1*	F: ATCACTCACTGTGCCAGAGC<br>R: CAGCGGTCTTGACGTTGTTG	103.98	113
XP_010742473.1	*ABCF2*	F: CGTAACGGAGCAGAGAGCAA<br>R: ATTAGCCCATAGCGTCTGCC	95.76	105
TMS14270.1	*FGF1*	F: TTCGTCCAAGCCTCAACCTC<br>R: TCTCGCCTTTGATGACCACC	98.11	133
KAG8004658.1	*TNFR5*	F: TCCGAGACTTTTCCTGGCTG<br>R: ACACTGGTCACAGCATAGCC	101.33	157
XP_010752206.1	*VEGFC*	F: CCAAACTGGAGACATGGGCA<br>R: CCAAACTGGAGACATGGGCA	104.21	168
XP_031133615.1	*BIP*	F: GCTGAGGCTTATCTGGGCAA<br>R: GCCACCACTTCAAACACACC	107.28	125
XP_041802934.1	*SPT3*	F: AGCGGATGGAGCGTTTAGAG<br>R: CTGGGCAACCGTCTCATAGG	106.80	180
TKS72241.1	*FRA1*	F: ACCTCCTTACCCAACCGTCT<br>R: GTTCCGGCGTCTTGTTGAAG	106.95	118
XP_010745692.1	*JUNB*	F: ACAAGCCTCTCTTCCGCATC<br>R: TCCTGGGTCTCCAAGTCGAT	102.26	105

Note: The mRNA sequences for each gene were obtained from *A. grunniens* transcriptome se-quencing database.
